# Does any aspect of mind survive brain damage that typically leads to a persistent vegetative state? Ethical considerations

**DOI:** 10.1186/1747-5341-2-32

**Published:** 2007-12-17

**Authors:** Jaak Panksepp, Thomas Fuchs, Victor Abella Garcia, Adam Lesiak

**Affiliations:** 1 Department of VCAPP, College of Veterinary Medicine, Washington State University, Pullman, Washington, USA; 2 Baily Endowed Chair of Animal Well-Being Science, Department of VCAPP, College of Veterinary Medicine, Washington State University, PO Box 646520, Pullman WA 99164-6520, USA

## Abstract

Recent neuroscientific evidence brings into question the conclusion that all aspects of consciousness are gone in patients who have descended into a persistent vegetative state (PVS). Here we summarize the evidence from human brain imaging as well as neurological damage in animals and humans suggesting that some form of consciousness can survive brain damage that commonly causes PVS. We also raise the issue that neuroscientific evidence indicates that raw emotional feelings (primary-process affects) can exist without any cognitive awareness of those feelings. Likewise, the basic brain mechanisms for thirst and hunger exist in brain regions typically not damaged by PVS. If affective feelings can exist without cognitive awareness of those feelings, then it is possible that the instinctual emotional actions and pain "reflexes" often exhibited by PVS patients may indicate some level of mentality remaining in PVS patients. Indeed, it is possible such raw affective feelings are intensified when PVS patients are removed from life-supports. They may still experience a variety of primary-process affective states that could constitute forms of suffering. If so, withdrawal of life-support may violate the principle of nonmaleficence and be tantamount to inflicting inadvertent "cruel and unusual punishment" on patients whose potential distress, during the process of dying, needs to be considered in ethical decision-making about how such individuals should be treated, especially when their lives are ended by termination of life-supports. Medical wisdom may dictate the use of more rapid pharmacological forms of euthanasia that minimize distress than the *de facto *euthanasia of life-support termination that may lead to excruciating feelings of pure thirst and other negative affective feelings in the absence of any reflective awareness.

## Introduction

In current medical practice, consciousness after brain damage is typically graded into 5 major categories: coma, persistent vegetative state (PVS), akinetic mutism, hyperkinetic mutism and delirium [1,2]. Coma and PVS are considered to constitute a brain state where the lights of consciousness have dimmed completely, usually temporarily in coma and typically forever in PVS during which, it is widely believed, only the control of bodily functions and unconscious neural processes continue in the brain. There are emerging reasons to re-consider such a conclusion.

Although modern neuroscience has by no means embraced the study of consciousness, it has become a major topic of discussion and empirical work in the larger arena of *mind sciences*; however scientifically solid insights and progress have been modest [3]. The topic largely remains a focus of philosophical discussion and speculation. At best there are suggestive findings, with nothing so definitive as to have garnered general agreement on how to measure consciousness, how many types there are, and how it is constituted within the brain. It is still commonly claimed that only humans are conscious, while most other animals are only complex neurobiological machines with no internal experiences. Indeed, some very bright people claim that even if consciousness exists in complex biophysical mechanisms like human brains, it may be epiphenomenal, having no causal efficacy in the control of behavior. Clearly, consciousness is a concept that remains in flux. All of these issues impact neurological practice that has to deal with impairments of consciousness, and it is truly a nightmarish ethical dilemma on how to treat people with seemingly totally impaired consciousness, when the possibility exists that fragments of consciousness remain.

We only highlight these issues to be clear, at the outset, that there is little agreement about issues and concepts in this area. Thus, to try to deal with a topic such as whether any consciousness remains in people who have descended into PVS cannot be definitively resolved by science right now. Hence, any attempt to discuss ethical issues that surround treatment of people in such states, will have to be an exercise in possibilities, based on the weighing of scientific evidence, where every finding has multiple interpretations. However, a discussion of possibilities is important for two reasons: 1) Society at large, and the legal system, are not as concerned with discussions about the existence and importance of consciousness in humans as are some philosophers and scientists. They simply accept the obvious conclusion, on *prima facia *evidence, that consciousness is an incredibly important part of each and every normal human life, and fall back on informed medical opinion, rather than science, on where we stand in this murky area of provisional knowledge. 2) Clearly an ethical society would treat people differently if they have any consciousness left than if they do not, which requires periodic re-evaluation of scientific progress and evidence in this arena. Thus, our discussion is premised on the acceptance of these framing issues as reasonable starting points for this inquiry into the ethical issues.

We will first present the most critical ethical issues as we see them, especially when it comes to the matter of whether to terminate medical life-support for these unfortunate individuals, and if that decision is made, what kind of guidelines should be considered in order to insure that individuals, in their passage toward bodily death, shall not be subjected to "cruel and unusual punishment"... something that we legally provide for convicted criminals, and surely would not deny unfortunate innocents.

We will highlight one major concern that should be foremost in peoples minds, but has received little attention in several recent high profile cases, like that of Terry Schiavo. The most important ethical issue as we see it, is whether someone like Ms. Schiavo, who had no apparent awareness of events around her, might still have had rudimentary emotional and bodily feelings in her uncomprehending mind. Eventually, at the behest of the legal system, artificial alimentation was terminated, and she was allowed to die from dehydration. The essential question, as we see it, is whether there is any good reason to believe that she may have experienced distressing, perhaps excruciating, pure thirst, without cognitive reflections, before she died. On the basis of current neuroscientific thinking, we believe an adequate case can be made for such a possibility. All the basic mechanisms of thirst exist in brain areas that typically remain undamaged in PVS. If there is reasonable scientific evidence to consider such possibilities – namely that affective feelings can exist within the brain without reflective awareness – then it is possible that Ms. Schiavo and others treated like her were subjected to "cruel and unusual suffering" by both legal and medical systems during their "natural" dying process.

We recognize that this is a very controversial perspective to an issue not yet resolved, so we will pursue the argument first by providing a synopsis of what we scientifically know about PVS. Following a description of the PVS condition, we will highlight why existing neuroscience data can presently support the possibility of a low level of consciousness, what we would call affective consciousness [4] still being operative in a brain that has succumbed to PVS. To anticipate our main point, we will argue that in a primitive unreflective state of affective consciousness, strictly in the phenomenal rather than self-reflective realm, one can feel pure pain, pure fear, pure anger, and other pure emotional feelings that are free of cognitive awareness. There are good reasons to believe such affective, raw experiential states can exist in the lower regions of human and animal brains when all higher substrates of mentality, especially of the neocortex, the widely accepted seat for all higher cognitive abilities including self-reflective experience and thought, is eliminated.

Although various kinds of brain damage can lead to PVS, it is generally agreed that if adult human beings were to lose all of their neocortex, they would fall into a persistent vegetative state. At the end of our description of PVS, we will summarize evidence for the conclusion that when such brain damage is inflicted on very young experimental animals, or when this type of damage occurs congenitally in human children, by all available external indicators, they are still capable of raw affective experiences, even though they no longer have any apparent thoughts or awareness of having such experiences. If our interpretation of these kinds of severe neurological conditions is correct, then both our medical and legal systems need to re-evaluate and perhaps modify how PVS patients deserve to be treated at the end of life.

### A short description of the persistent vegetative state

In 1940 Ernst Kretschmer described a condition he named "apallic syndrome" a state "without cortex". Twenty-three years later, Arnaud and colleagues [5] coined the phrase *vie vegetative*, to describe a subset of patients with severe head injury. It was not until 1972 that Jennett and Plum attempted to describe the clinical features of what is now known as the vegetative state (VS). Consequently, the same authors proposed the concept of a Persistent Vegetative State (PVS) to distinguish patients who remain in a PVS from those who ascend through a vegetative state from coma to consciousness. The year 1982 brought a further distinction between "persistent" and "permanent" vegetative state (pVS; [6]), based on the very low probability of recovery from a PVS that lasts longer than 12 months. In 1991 a Multi-Society Task Force on PVS (MSTF) was established with the goal to provide guidelines (clinically and ethically) for diagnosis and treatment of this syndrome. The resulting two articles published in The New England Journal of Medicine [7] are currently considered the authoritative voice on this condition. PVS as it is understood today is characterized by 7 diagnostic criteria:

"1) No evidence of awareness of self or environment and an inability to interact with others. 2) No evidence of sustained, reproducible, purposeful, or voluntary behavioral responses to visual, auditory, tactile, or noxious stimuli. 3) No evidence of language comprehension or expression. 4) Intermittent wakefulness manifested by the presence of sleep-wake cycles. 5) Sufficiently preserved hypothalamic and brain-stem autonomic functions to permit survival with medical and nursing care. 6) Bowel and bladder incontinence, and 7) Variably preserved cranial-nerve reflexes [...] and spinal reflexes." (Task-Force, page 1501)

A PVS can arise for a number of reasons, and the clinical course of this state depends on the particular underlying disorder. There are three main categories of neural injury that can lead to a PVS:

1) Acute Traumatic and Nontraumatic Injuries: The most common acute causes of a PVS are head trauma and hypoxic-ischemic encephalopathy. It is rather difficult to outline a common clinical course for these cases but the PVS is usually preceded by a coma with a certain number of these patients (1–14%) their condition develops into a PVS. To date it is not entirely clear which properties of a coma predict a vegetative outcome; however the presence of ventilatory dysfunction, decorticate posturing, and extraneural trauma soon after the insult are potential indicators. Other correlates include advanced age, pupillary abnormalities, and a low motor response score. In nontraumatic comas an impairment of eye opening, the presence of abnormal oculocephalic, abnormal motor responses, and the inability to obey commands after two weeks are all correlated with a vegetative outcome.

2) Degenerative and Metabolic Disorders: Various degenerative and metabolic nervous system disorders in adults and children inevitably progress into an irreversible vegetative state.

3) Developmental malformations: The immaturity of the developing brain at early ages presents a considerable challenge for the diagnosis of a PVS in infants and children. Most of the criteria used for diagnosis are not applicable to patients less than 3 months old. In these patients it is difficult to distinguish between voluntary and involuntary responses, or to detect sleep-wake cycles.

### Neuropathology and the persistent vegetative state

Due to the various etiologies, it has proved difficult to define PVS based simply on a shared neuropathology. Yet, over the years, certain communalities have emerged that are consistent with the idea that PVS, more or less, represents a state of functional decortication. One of the first attempts to assess the underlying neural basis of the persistent vegetative state was made by Kinney and Samuels [8]. In their review they proposed three characteristic patterns of brain damage associated with a vegetative state. These patterns involved damage to the cerebral cortex, widespread damage to the white matter of both hemispheres, and damage to the thalamus.

The Task-Force on PVS [7] identified two main sources of Brain damage in PVS patients:

1) Diffuse laminar cortical necrosis, which is typically a result of global hypoxia and ischemia. Extensive multifocal cortical necrosis that almost invariably involves the hippocampus and may be accompanied by damage to hypothalamus, and areas of the brain stem.

2.) Diffuse axonal injury (DAI) is due to a shearing injury as a result of acute trauma. The resulting axonal damage isolates the cortex from other parts of the brain. Occasionally DAI is also accompanied by small lesions in the brainstem.

When Kampfl and colleagues [9] conducted a magnetic-resonance imaging (MRI) study on 80 patients in a vegetative state they found damage in the corpus callosum, the main connection between brain hemispheres, in 98% of the patients, and also detected injuries to the white matter of the frontal and temporal lobes. Lesions in both corpus callosum and the dorsolateral brainstem were described as the most frequent combination in this subset of PVS patients.

More recent post mortem studies on the anatomical basis of PVS [10] detected DAI in 70–80% of the studied brains. Other common features included abnormalities in the thalamus in near 80% and ischemic damage in close to 40% of the patients.

Giacino & Whyte [11], identified two patterns of brain damage in a VS. The most common pattern is Diffuse Axonal Injury (DAI) associated with moderate to severe ischemic damage that usually involves the thalamus. More recently, Graham and colleagues [12] described neurodegenerative changes in the thalamus and hippocampus associated with this pattern. A second less common pattern leading to a vegetative state is characterized by focal brain stem lesions, not accounted for by DAI.

In essence all of these findings support the conclusion that neural damage either to large cortical regions (hypoxia/ischemia), or to brain areas connecting cortex and brain stem (thalamus), as well as damage to the connections between cortical areas (corpus callosum, thalamo-cortical loops, DAI) can lead to a PVS. Therefore the old notion of an "apallic state," reflecting a functional disconnect of cortex and brain stem can still be maintained. It should also be emphasized that severe damage of the brainstem seldom occurs in PVS patients [7] and that brainstem damage alone, even if initially provoking coma, rarely results in a vegetative state [13].

### Neuroimaging of PVS

In current clinical practice, the diagnosis of a persistent vegetative state does not fully utilize neuroimaging techniques, but relies heavily upon bedside clinical evaluation. The use of electroencephalogram (EEG) and functional neuroimaging techniques cannot fully replace the current techniques used to diagnose patients with consciousness disorders; however they can potentially aid practitioners in identifying the specific traits of the patients' injury and in forming a better rehabilitation strategy. In the following we provide a brief overview of the properties of PVS when examined with electrophysiological and brain imaging techniques with some emphasis on the question if such measures could provide some evidence for the presence or absence of phenomenal experience, perhaps even some awareness, in a state that, by definition, excludes the possibility of conscious experience.

### Electrophysiological measures of brain activity

The EEG is a measure of cortical activity that is widely used to study arousal states, most prominently sleep wake cycles, epileptic seizures, or the actions of drugs on the brain. In clinical practice it is also used to determine brain death (i.e. an absence of cortical brain activity signified by an isoelectric EEG), and therefore has ramifications for the legal definition of death.

The EEG of healthy subjects can be interpreted as a reliable indicator of attention and arousal states. For instance, in healthy subjects, a mixed frequency low amplitude (desynchronized) EEG accompanies alert wakefulness while a synchronized alpha rhythm (8–13 Hz) that is suppressed by light (vision-induced alpha blocking) is characteristic for a state of relaxed wakefulness. Rapid eye movement (REM) sleep is characterized by an EEG similar to alert wakefulness, while synchronized high amplitude theta (4–8 Hz) and delta (1–4 Hz) waves are found during the various stages of non-REM sleep. Also, sustained higher frequency rhythms (beta and gamma) in the cortical EEG have received considerable attention as potential correlates of consciousness (e.g. [14]).

When considering the cortical EEG of PVS patients we have to keep in mind that:

1) PVS is a state, not a disease. It is the endpoint to many pathologies. Consequently, and not surprisingly, the EEG of PVS can vary considerably among patients.

2) PVS is entered through a coma. Electrophysiologically PVS and coma represent a continuum.

3) PVS is a long term but variable brain state and, over time, the properties of the EEG may change [7]. During a slow recovery from traumatic injury some properties of a healthy EEG may return over time while the patient, nonetheless, remains in a VS.

Many PVS patients show an EEG of "diffuse generalized polymorphic delta or theta activity" [7]; generally, a slowing of EEG background activity is associated with alterations of consciousness, stupor and coma [15]. Some patients display an almost isoelectric EEG while the brain activity of other patients is characterized by a continuous non-responsive alpha rhythm. In a few patients the EEG is reported to be near normal, with the exception of vision-induced alpha blocking [7,16]. Also, EEG patterns do not change in response to stimulation with the interesting occasional exception of noxious stimulation [7]. Such EEG properties are shared by many comatose and even minimally conscious state (MCS) patients. Gamma activity has been found to be present in all tested PVS patients [17], however, an increase of gamma power together with conjugate eye movements, a sign of voluntary movement in healthy subjects, was only seen in "less severe" cases [17]. To our knowledge, only one EEG study (quantitative electric tomography, QEEGt) detected specific activity changes in the EEG spectrum of an 8 year old PVS patient in response to his mothers voice [18]. However single case studies, especially in a state with such an obvious potential for misdiagnosis have to be interpreted with caution (also see below).

Considering the history of the EEG, its wide use in sleep research, and the very definition of human sleep as a combination of electrophysiological and behavioral parameters one would be inclined to believe that the clinical definition of "sleep-wake cycle" in PVS should rely on EEG measures; however, this is not the case. The definition of sleep in PVS is entirely behavioral. The patient will show periods of behavioral inactivity with closed eyes that are followed by periods of (non purposeful) behavioral activity with open eyes. An EEG study on sleep in PVS patients revealed that the EEG during this behavioral sleep-wake cycle can be quite variable [19]. In one patient no cortial surface EEG changes between "sleep" and "wakefulness" were observed, in some an EEG of low amplitude delta and theta waves were observed during behavioral sleep. In two of these patients the slow wave activity increased over time. None of the patients showed consistent sleep staging, and EEG indicators of REM sleep were entirely absent. In contrast other studies were able to identify behavioral indicators of REM sleep (myoclonus and rapid eye movements) in PVS patients [20]. One could therefore interpret these findings in a way that the midbrain/pontine and hypothalamic sleep/wake generators are functional and active. However, because in most cases of PVS the connectivity between lower brain areas and cortex is diminished, cortical EEG activity does not show the full range of changes associated with normal sleep.

Event related potentials (ERPs), a derivate of the EEG, have shown some promise in investigating cognitive processing and possibly conscious awareness in PVS patients. ERPs are small positive and negative deflections of the EEG that occur time-locked to specific (commonly auditory) signals. ERPs are characterized by positive (P) and negative (N) deflections of early, middle and late components which are believed to contain information about signal processing. Late components are typically associated with higher level cortical processing and correlate in some cases with the presence or absence of conscious experience. This makes them a potential tool in the assessment of awareness.

ERP studies have provided some evidence of higher order cortical processing in PVS patients. The N1–P2 complex, an indicator of low level cortical processing has been detected in most PVS patients [16]. Mismatch negativity (MMN) and P3, ERP components accompanying higher order processing have also been detected in a number of patients [21]; however, none of these components are clear indicators of conscious awareness. Nevertheless, the MMN an evoked response to stimulus deviation seems to be of particular value in predicting the outcome of PVS [16,22]. Its presence during the acute phase of coma allows for exclusion of PVS as a possible endpoint [22], and its occurrence in PVS patients predicts recovery from their current state [22-24]. The late ERP component "pain-related P-250" has been found to be greatly depressed [25] in vegetative states, therefore PVS patients may not be aware of pain. Interestingly, Katayama and colleagues [25] also demonstrated that deep brain stimulation can restore the P-250 in some PVS patients.

### Functional neuroimaging

With the development of functional neuroimaging techniques such as positron emission tomography (PET) scans and functional magnetic resonance imaging (fMRI), new insights into the nature of the persistent vegetative state have been gained.

PET scans of patients in a vegetative state consistently show a reduction in cerebral metabolism with lower metabolic activity associated with the duration of a vegetative state [26]. Laureys et al. [27] showed major impairment of the prefrontal, premotor and parietotemporal association areas, as well as in the posterior cingulate cortex/precuneus in their study of the vegetative state using PET scans. This correlates the vegetative state with a cortical disconnect between association cortices, rather than structural cortical damage [26,27]. Moreover, other studies have shown that in addition to altered cortical connectivity, the corticothalamic system may also be deficient [28].

### Functional neuroimaging of sensory processes

Studies have used functional neuroimaging to look for the subsistence of sensory and perceptual systems in people with consciousness disorders with the intent to elucidate if the patients retain some form of awareness. Visual fixation and tracking is a property seen in a minimally conscious state; however the extent of the patient's awareness of stimuli is unknown because the patient is unable to articulate a response. It is possible that these visual actions may represent involuntary responses devoid of cognitive properties ([26], see also EEG above). Studies using PET scans to observe patients' response to flashes of light showed consistent activation of primary visual cortex; however a further activation of higher-order association areas was not detected [26].

Similar to studies of visual stimulation, simple auditory stimuli resulted in an activation of primary sensory cortex, but failed to reach higher order processing areas of the posterior parietal cortex, anterior cingulate cortex, and hippocampus [26]. Further evidence for the lack of higher-order processing of sensory stimuli in a vegetative state can be seen in a study investigating brain activation in response to noxious somatosensory stimuli. The responses of patients in a vegetative state in response to high-intensity median nerve stimulation at levels previously shown to be painful in control subjects were recorded using PET scans. This study showed activation of primary somatosensory cortex as well as midbrain and thalamus activity in all patients, yet with a lack of associative cortical activation, similar to the results of other sensory studies in PVS patients [29].

In summary, functional neuroimaging studies of sensory processing in patients suffering from disorders of consciousness strengthen the theory of a cortical disconnect in patients in a vegetative state. Studies investigating visual, auditory, and somatosensory processing all showed that PVS patients retained some activation of primary sensory cortex areas without further processing in nearby association areas [26]. In short, sensory signals in these patients reach the cortex, but the extent to which these signals are experienced a primary-process perception remains unclear.

### Learning in a persistent vegetatives state

The evidence of cortical learning in a vegetative state is limited. Indeed, few controlled studies on this subject have been performed. Koutchoubey and colleagues [30] report habituation of the blink reflex in some PVS patients, and also demonstrated that the cortical ERP component N1 shows habituation over time, indicating some continuation of both subcortical and cortical learning process. Accordingly, additional conditioning studies should be conducted with emotionally provocative stimuli to determine how much plasticity, especially of emotional capacities, persist in PVS.

### Possible conscious awareness in PVS

A recent fMRI study of a clinically diagnosed PVS patient showed specific brain activation with the administration of two different spoken instructions. In one instance the patient was asked to imagine playing tennis, while in the other the patient was instructed to imagine visiting all the rooms of their house. Brain activity changes were congruent with those observed in controls under both conditions [31].

While it is possible to entertain the idea that this study shows strong evidence for conscious awareness in the patient, the brain activation may have been an automatic response to the presented narrative [32]. Although the study does not indicate any conscious "decision to cooperate" in the subject, it does highlight potential shortcomings of common clinical diagnosis. According to the authors, the patient examined met all clinical requirements of PVS. However, the observed brain activation in response to the instructions suggests either a misdiagnosis or some higher information processing capabilities in a PVS state. A misdiagnosis seems more likely, considering that a single patient was examined. The fMRI data may indicate that this patient may have progressed into a minimally conscious state distinguished by the ability to follow commands while still lacking the capacity to communicate [33].

### Imaging techniques and consciousness

Can the techniques of modern neuroscience provide us with a *definitive *answer as to whether patients in vegetative states retain some remnants of phenomenal experience or not? The simple answer is no, not presently. However, only a few years ago it was equivalent to heresy to mention consciousness and neuroscience within the same breath, let alone to attempt scientific research on the subject. The study of awareness and the neural correlates of consciousness are new branches of experimental neuroscience and although some promising findings encourage future research into remnants of phenomenal experience in PVS, the present state of knowledge allows for hypotheses at best, not for any differential clinical diagnosis. Also we do not want to imply that the current predominantly behavioral methods of diagnosing disorders of consciousness are fully acceptable. In both cases the diagnosis relies on correlates-behavioral or neural correlates. Such correlates may be sufficient to avoid a misdiagnosis in the strict sense, to distinguish e.g. between a locked in syndrome and a PVS. The root of the problem, however, is the question whether a properly diagnosed PVS patient may have some remnants of phenomenal sensory or affective experiences. These problems cannot be addressed by behavioral measures, while imaging techniques, at present, are not fully developed to address such questions. This leaves us with some enormous ethical dilemmas.

Historically, it has been often assumed that conscious experience is solely a property of the neocortex. In our estimation, there is little empirical support for such an extreme corticocentric view of consciousness. There really is no well-conceived reason as to why subcortical brain structures should not be able to generate unreflective affective states and basic experiences of homeostatic drives such as thirst or hunger, and abundant research in animal models that has worked out some of the details of such brain processes (see below) strengthens this assumption [34-36]. The fact that in PVS patients damage to cortex and thalamus is more pronounced than damage to lower brain areas strongly suggests that such raw affective states could still be experienced by patients in a vegetative state.

### Affective vs. Cognitive Consciousness

When it comes to understanding consciousness, definitional problems and disagreements can abort clear communication. Although there is a very rich conceptual literature on these topics which cannot be covered here [3], and various distinct types of consciousness have been proposed, the present discussion will rely on the distinction between affective and cognitive consciousness that has been developed by the senior author [4,34,35,37].

A functional evolutionary view of phenomenal consciousness, namely the here and now experience provided by outward sensory portals as well as the intrinsic functions of the brain mind, suggests there are ancient, affective forms of consciousness that emerged long before organisms developed any sophisticated cognitive abilities such as the capacity to reflect on ones experiences, and to know one was conscious. Current knowledge of the brain strongly supports the idea that primary-process affective experiences emerged in brain evolution earlier than those high cognitive processes that allow us to think and talk about our internal experiences (i.e., secondary and tertiary forms of consciousness). In all mammals, the neural systems that are essential for emotionality are concentrated in more medial and ventral brain regions than most cognitive processes. These ancient systems seem to be the epicenters for emotional and other basic affective experiences such as the pleasure of taste and the distress of homeostatic imbalances from air-hunger to thirst [36].

Although no one has decoded how affective experience is constructed from neural activities, it is clear that there is a complex instinctual-emotional brain situated in deep subcortical regions. This is simply demonstrated by the ability to provoke a variety of coherent emotional behaviors using localized electrical stimulation of the brain (ESB) applied to those circuits [34,35,37]. One can also ask animals whether they like, dislike or feel neutral about such stimulation. They are rarely uncertain; they turn on ESB that seems to evoke positive external appearances, such as foraging, sexuality, and predatory behaviors, and they turn off ESB that evokes emotions that most would consider aversive, such as fear and anger. A remarkable aspect of these studies is that the affective intensity increases as one stimulates ever lower reaches of these systems, as in the periaqueductal gray, where emotional responses and feelings (in both humans and animals) are provoked by the lowest levels of ESB [37].

Since the "center of gravity" for emotional feelings resides in more ancient parts of the brain than most cognitive functions, we can surmise that those brain regions have a primitive affective-experiential mind of their own. How can this be evaluated? The clearest evidence comes from radical decortication when animals are very young. Abundant work has been done with such preparations, especially in laboratory rats, and the general principle is that these animals look very normal, exhibiting all of their instinctual emotional tendencies, including playfulness [38]. Indeed, they show improved behavior in certain difficult learning tasks such as two-way avoidance (shuttling between two successively dangerous areas of a test box), where normal animals get befuddled and apparently so intimidated that their immobility prevents efficient behavior. Decorticate animals, being generally disinhibited, shuttle readily, probably with no awareness of the benefits their tendencies for over-activity are producing for them (Panksepp, unpublished personal observation). These animals are also not as perceptually impaired as one might expect from studies of animals decorticated at an older age when cortical functions have matured [39].

If animals can function so effectively without their cortices, how about humans? Obviously, just as in other animals, decortication after maturity dramatically deteriorates cognitive ability, to the point where such humans would typically descend into PVS. However, this is not the case with infants that are born without their higher brain apparatus, especially if they have been reared in a loving environment with abundant, emotionally supportive experiences [40]. Such children seem to experience the world affectively and perceptually, but they have no way to tell anyone about their experiences. That must be inferred from behavior. This is a dilemma, for one can have no certainty of the level of their experiential capacities, but when the external manifestations are intact, and when the relevant brain science, supports a dual-aspect monism view of how emotional feelings emerge from neural circuits [35,41], then the weight of evidence supports that such creatures do have a level of mentality that deserves our attention and respect. Dual aspect monism in affective neuroscience reflects the viewpoint that primary-process psychological processes, such as affect, arise from brain dynamics that are a real part of nature and that such evaluative feelings are isomorphic with certain types of large scale neural network dynamics that are strongly linked to inherited brain-mind potentials. Various instinctual feelings seem to arise from genetically provided circuits within ancient regions of the mammalian brain.

The common failure not to distinguish among the evolutionary layers of consciousness can lead to many conceptual conundrums and communicative confusions. For instance, some emotion researchers view emotional experience as reflecting the highest neocortical reaches of the human brain [42,43], while our reading of the evidence is that raw affective experience is almost completely a sub-neocortical experience [37,41]. Obviously, such investigators are talking about different evolutionary levels of consciousness, and most seem to put more stock on the cognitive awareness aspects than the more primitive affective state functions that may not be intrinsically accompanied by self awareness.

Thus, the present analysis is based on the primal assumption that the raw phenomenal experience of organisms living in highly variable environments reflects the most important survival-mediating, value-generating structures of the brain.

In our estimation, affective consciousness, perhaps the most ancient variant (think of pure *pain*), arises from the capacity of our brain to experience the biological values of the body – organismic conditions that can unconditionally enhance or detract from survival. Cognitive consciousness – the ability to discriminate multifarious differences in the world – seems not to be foundational in the capacity of the brain to have raw affective experiences.

Do PVS patients still have remnants of affective consciousness? We cannot be certain, but we can be sure they still exhibit a variety of instinctual behaviors, such as apparent anger attacks, that may not simply be "sham rage" accompanied by no internal feelings states. If raw emotional feelings are created by such brain activities, we should find no comfort in the fact that we have simply defined away the potential existence of raw affective experiences within the mental remnants of such PVS patients.

### Raw Affective Experience in PVS and Emerging Ethical Issues

The PVS state and how various social groups in our culture respond to such helpless patients are pregnant with ethical ambiguities, especially so once the withdrawal of life-support is considered. Although, all we can do is consider possibilities, with no definitive prescription, we take as our categorical imperative that all quarters of society, whether governmental, medical, religious, or every-day people, should minimize the likelihood of suffering during the end of life, whether it be "natural" or induced.

We acknowledge that there can be considerable debate about whether suffering can exist without reflective awareness. We believe it can, especially when one recognizes that the aversive component of suffering arises largely from an intensely negative affective state, and that under normal circumstances, cognitive activity, if anything, tends to reduce the intensity of experienced affect [44]. Thus, the sustained persistence of excruciating negative affect, in the absence of reflective awareness, may be a terrible experience.

At the point where termination of life support is considered, once any reasonable medical expectation of functional recovery has dimmed, there is a shift in the equation from the possibility of inflicting "suffering," in the hope of obtaining recovery, to inflicting "cruel and unusual suffering/punishment" (as imposed "suffering," when hope of recovery has been relinquished, may become isomorphic with "punishment"). In the absence of definitive knowledge about the affective suffering of PVS patients, something that is not empirically achievable at present, "intent to cause suffering" (i.e. punishment) only becomes part of the overall equation, depending on whether one believes that there is currently enough "weight of evidence" to be concerned about the potential for primary-process negative affective states in PVS patients. We believe such evidence now exists (previous section). Even though the 1994 Task-Force report on PVS [7] did raise the topic of "pain and suffering," without considering other highly aversive states such a thirst, sustained cramps and many other bodily discomforts, they concluded that since these patients were "unconscious by definition" that "precludes these experiences."

We believe new knowledge and evolutionary perspectives do *not *preclude raw, unreflective affective experiences in the mental residue of PVS patients. Indeed, the PVS Task-Force [7], recognized that in PVS patients "subcortical nociceptive responses produce patterned behaviors, such as grimace-like or crying-like behavior similar to that accompanying conscious emotional responses." However, they concluded that since "conscious awareness of pain or the experience of suffering occurs at a cortical level" the instinctual behavioral indices of pain do not actually reflect the experience of any pain. Regrettably, the Task-Force did not attempt to make any distinction between the ability of an individual to be *aware *of one's pain, and the ability to experience a purely unreflective form of pain. PVS patients also show seemingly painful cramps, "sham" rage attacks, and other instinctual indices of emotional states. As summarized in the previous section, the neuroscientific evidence now strongly suggests that raw emotional experiences arise, in part, from those same brain systems that mediate emotional-instinctual responses [34,35,41]. There is currently no compelling evidence that raw *emotional *feelings emerge through some kind of a "read-out" processes whereby higher neocortical processes that mediate awareness of the world reinterpret lower instinctual brain functions.

The medical system has traditionally chosen to make the concept of cognitive "awareness" an essential, if not the sole, conceptual criterion of consciousness. By doing so, they have, simply by conceptual definition, eliminated affective experience from the potential residual mentality of PVS patients. By siding with the *assumption *that *all *aversive experiences require cortical participation, they have generated a practical consensus policy on how to conceptualize PVS. However, the concept of a primitive affective phenomenal consciousness provides an alternative perspective that now mandates a reconsideration of what is and what is not supported by existing evidence. To the extent that ambiguities remain, policy should dictate that patients are insured absence of any primary-process agony as much as possible.

If we consider the accruing evidence and theory about the basic nature of affect, and use it as a guideline for our thinking, we would be wise to accept the realistic *possibility *(although perhaps not the high *probability*, as we do) that PVS patients can still experience some remnants of affective experience even though their cognitive abilities are gone. If we do, there are some clear ethical consequences that need to be considered by all quarters of society, regardless of ones cultural, political or religious dispositions.

The moment we acknowledge the possibility that an individual in a vegetative state may experience raw affective experiences, the mere discontinuation of life support in the form of food or drink cannot be a viable strategy, if the goal remains to "do no harm." We are now confronted with the possibility that the patient, on her/his passage to death, is experiencing excruciating feelings of hunger, thirst or pain sometimes for many days. An inability to outwardly communicate this suffering may be emotionally comforting to family members and medical personnel, but this only underscores once more how much our everyday definition of wellbeing depends on coordinated behavioral output, a route that is cut off for patients in a vegetative state. Parenthetically, it should be mentioned that similar issues are being debated in the criminal justice system, where the use of muscular paralytics during lethal injections, denies the condemned the possibility of communicating their suffering, no matter how short that may be (as we write this, the United States' Supreme Court has agreed to reconsider this issue).

Consequently, to ensure that patients will not be exposed to feelings of discomfort (or to limit feelings of discomfort to as short a period as humanly possible) it becomes necessary to either supplement the discontinuation of life-support with pharmacological means that are most likely to alleviate any distress, as might be achieved with mixed opiate agonists/antagonists such as buprenorphine, or to provide a form of active euthanasia (e.g. a full opioid overdose to make the passage to death as rapid and bearable as possible). We realize and respect that today, for many members of society and for a variety of reasons, such an act is entirely unacceptable. In the end, we believe that such decisions should always be left to the patient (if possible) or the patients family (supported by professionals). Indeed, if constrained only by logic, active euthanasia seems a viable strategy considering that, if our hypothesis is wrong and PVS patients lack conscious experience no additional harm is done by e.g. an overdose of drugs that can promote and sustain positive affect. On the contrary, if we were only right in a single case the individual would benefit enormously. However, it is not the purpose of this article to make a strong case for discontinuation of life support, merely to bring to attention what needs to be considered if it is entertained as an option.

It seems plausible that the possibility of raw affective experience in a PVS state may sway some individuals or institutions to take an even firmer stance against all forms of discontinuation of life support, whether passive or active euthanasia. Despite this dilemma, we do need to recognize the potential of these patients to *experience *something. There is probably general agreement that we should aim to make these experiences as positive as possible. Of course, indefinite continuation of life support would lead into immense financial burdens on individuals, certain institutions and society as a whole. But we believe costs should not figure as a priority in an ethical discussion, and we do not believe that indefinite continuation of life-support is the only ethical option. At times like this, quality of life issues loom large.

In any event, to fully evaluate the possibility that PVS patients may have residues of affective consciousness in their uncomprehending minds, we would urge the medical community to support additional research on this topic. A variety of novel behavioral tasks, such as the classical conditioning of emotional responses, in conjunction with the new brain imaging technologies, deserve to be implemented more vigorously. The work of neurologists and their ethical responsibilities are complete when they have done everything currently possible to restore higher forms of consciousness in such individuals using accepted medical approaches, which in the future are likely to include new neuropharmacological modalities, and perhaps localized stimulation of subcortical networks that have some potential to restore higher levels of mentation. When doctors' skills and options have been exhausted, their only remaining dictum is "do no harm." The mere disconnection of life support does not ensure that.

Although ethics/morality is impossible to prescribe, we would suggest that our knowledge of how brain functions generate raw emotional feelings, has reached a point where the existence of strong feelings in brains, without any remaining cognitive reflective capacity, is substantively supported by our current understanding of how brains operate. If so, such possibilities should figure heavily in our ethical discussions of how to handle PVS individuals when termination of life-support is considered.

We recognize that our arguments are premised on the current weight of evidence from functional neuroscience rather than definitive facts. However, if raw feelings can remain in the residual brain functions of PVS patients, especially aversive affective ones, and if we do not recognize those possibilities, we are in a position to make enormous ethical blunders. Assuming all ethics ultimately are based on the intrinsic values that give us various feelings of *goodness *and *badness*, as some philosophers like David Hume have postulated, we must consider the various possibilities that a raw affective consciousness perspective offers for our ethical considerations [34,45].

The issue of what we, as a society, should do with someone who has a corner of consciousness left, even if we cannot detect it in their outward communications, is of momentous importance for any coherent vision of medical, governmental and religious ethics. We believe that in such existential circumstances, individual rights should prevail over all others. In a kind and just society, the notion of permitting any "cruel and unusual suffering" is so offensive that to impose "natural death" by simply withdrawing life-supports, namely a form of slow *de facto *euthanasia, with all the potential agonies of sustained thirst, hunger and various emotional and bodily pains, over a kind and rapid euthanasia, seems unreasonable. If affective consciousness can exist without cognitive capacities, removing life supports without providing graceful alternatives opens up the possibility of inflicting too many innocent people with a series of terrible feelings that they are in no position to alleviate. If this actually does happen in those unfortunates where courts have accepted the mere termination of life-support, even against family wishes, we may be condoning social practices that may be inflicting cruel and unusual punishment/suffering on some of the most helpless members of society. No human should be subjected to that. We do need a new round of discussions on what it truly means to be unconscious not just from an "awareness" point of view, but also from a raw "phenomenal experience" perspective.

In sum, although PVS raises a host of difficult medical, social, scientific and ethical issues, they can no longer be adequately addressed without a frank confrontation with the issue of how raw affective experience is created in the brain. How we ethically treat patients who have succumbed to PVS, especially when termination of life-support is considered, will eventually need to be critically informed by our emerging knowledge of what it means to have highly aversive affective experiences that may exist without reflective awareness. The mere termination of "life support" may lead to needless suffering, and that should be avoided.

## NOTE

As pointed out by one of the reviewers (Joseph Verheijde) an increasing shortage of organ transplants has resulted in attempts to utilize PVS patients as potential organ donors. Albeit inherently problematic [46,47], at present, the dead donor rule (DDR) and the increasingly practiced donation after cardiac death (DCD), protect patients in a PVS from organ procurement. In other words, PVS patients do not meet the criteria for brain death (intact brain stem) and therefore are typically able to autonomously maintain cardiac activity. Proposed changes to the definition of death or a departure from the DDR [47,48] could however change this situation. A plausible scenario would then include PVS patients into a category described as "higher-brain death" [48] which could likely result in organ procurement under brain dead protocols, i.e. *without anesthesia *(Verheijde, personal communication). The idea of redefining brain death as brain failure was discussed at the President's Council on Bioethics on September 6, 2007 [49]. The Council discussed classifying individuals who are unable to interact with the environment as having brain failure and therefore such persons are to be considered dead. The rethinking of brain death criteria is motivated by the need to increase opportunities for organ procurement, but the issue of pure affective experience must loom large as society considers pursuing that course of action.

## Competing interests

The author(s) declare that they have no competing interests.

## References

[B1] Schiff ND, Velmans M, Schneider S (2007). Global disorders of consciousness. The Blackwell Companion to Consciousness.

[B2] Watt DF, Pincus DI, Panksepp J (2004). Neural substrates of consciousness: Implications for clinical psychiatry. Textbook of Biological Psychiatry.

[B3] Velmans M, Schneider S, eds (2007). The Blackwell Companion to Consciusness.

[B4] Panksepp J, Ellis R, Newton N Affective consciousness and the instinctual motor system: the neural sources of sadness and joy. The Caldron of Consciousness: Motivation, Affect and Self-organization, Advances in Consciousness Research.

[B5] Arnould M, Vigouroux R, Vigouroux M (1963). Etats frontiers entre la vie et la mort en neuron-tramatologie. Neurochirurgica (Stuttg).

[B6] Plum F, Posner J (1982). The Ddiagnosis of Stupor and Coma.

[B7] Multi-Society Task Force on PVS (1994). Medical aspects of the persistent vegetative state. N Engl J Med.

[B8] Kinney HC, Samuels MA (1994). Neuropathology of the persistent vegetative state: A review. J Neuropathol Exp Neurol.

[B9] Kampfl A, Schmutzhard E, Franz G, Pfausler B, Haring HP, Ulmer H, Felber S, Golaszewski S, Alchner F (1998). The persistent vegetative state after closed injury: clinical and magnetic resonance imaging findings in 42 patients. J Neurosurg.

[B10] Graham DI, Adams JH, Murray LS, Jennett B (2005). Neuropathology of the vegetative state after head injury. Neuropsychol Rehabil.

[B11] Giazino J, Whyte J (2005). The vegetative and minimally conscious states: Current Knwoledge and remaining questions. J Head Trauma Rehabil.

[B12] Graham DI, Maxwell WL, Adams JH, Jennett B (2005). Novel aspects of the neuropathology of the vegetative state after blunt head injury. Prog Brain Res.

[B13] Parvizi J, Damasio A (2001). Consciousness and the brainstem. Cognition.

[B14] Sauvé K (1999). Gamma-Band synchronous oscillations: Recent evidence regarding their functional significance. Conscious Cogn.

[B15] Brenner RP (2005). The interpretation of the EEG of stupor and coma. Neurologist.

[B16] Kotchoubey B, Lang S, Mezger G, Schmalohr D, Schneck M, Semmler A, Bostanov V, Birbaumer N (2005). Information Processing in severe disorders of consciousness: Vegetative state and minimally conscious state. Clin Neurophysiol.

[B17] Balazs S, Stepan C, Binder H, von Gizycki H, Avitable M, Obersteiner A, Rattay F, Selesnick I, Bodis-Wollner I (2006). Conjugate eye movements and gamma power modulation of the EEG in persistent vegetative state. J Neurol Sci.

[B18] Machado C, Korein J, Aubert E, Bosch J, Alvarez MA, Rodriguez R, Valdes P, Portela L, Garcia M, Perez N, Chinchilla M, Machado Y, Machado Y (2007). Recognizing a mother's voice in the persistent vegetative state. Clin EEG Neurosci.

[B19] Isono M, Wakabayashi Y, Fujiki M, Kamida T, Kobayashi H (2002). Sleep cycle in patients in a state of permanent unconsciousness. Brain Inj.

[B20] Oksenberg A, Gordon C, Arons E, Sazbon L (2001). Phasic activities of rapid eye movement sleep in vegetative state patients. Sleep.

[B21] Perrin F, Schnakers C, Schabus M, Degueldre C, Goldman S, Bredart S, Faymonville ME, Lamy M, Moonen G, Luxen A, Maquet P, Laureys S (2006). Brain response to one's own name in vegetative state, minimally conscious state, and locked-in syndrome. Arch Neurol.

[B22] Wijnen VJM, van Boxtel GJM, Eilander HJ, de Gelder B (2007). Mismatch negativity predicts recovery from the vegetative state. Clin Neurophysiol.

[B23] Kotchoubey B (2007). Event-related potentials predict the outcome of the vegetative state. Clin Neurophysiol.

[B24] Daltrozzo J, Wioland N, Mutschler V, Kotchoubey B (2007). Predicting coma and other low responsive patients outcome using event-related brain potentials: a meta-analysis. Clinic Neurophysio.

[B25] Katayama Y, Takashi T, Takamitsu Y, Teruyasu H, Shuhei M, Seigou K (1991). Characterization and modification of brain activity with deep brain stimulation in patients in a persistent vegetative stat: pain-related late positive component of cerebral evoked potential. PACE.

[B26] Giacino JT, Hirsch J, Schiff N, Laureys S (2006). Functional neuroimaging applications for assessment and rehabilitation planning in patients with disorders of consciousness. Arch Phys Med Rehabil.

[B27] Laureys S, Goldman S, Phillips C, Van Bogaert P, Aerts J, Luxen A, Franck G, Maquet P (1999). Impaired effective cortical connectivity in vegetative state: Preliminary investigation using PET. Neuroimage.

[B28] Schiff ND (2005). Modeling the minimally conscious state: measurements of brain function and therapeutic possibilities. Prog Brain Res.

[B29] Laureys S, Faymonville ME, Peigneux P, Damas P, Lambermont B, Del Fiore G, Degueldre C, Aerts J, Luxen A, Franck G, Lamy M, Moonen G, Maquet P (2002). Cortical processing of noxious somatosensory stimuli in the persistent vegetative state. Neuroimage.

[B30] Kotchoubey B, Jetter U, Lang S, Semmler A, Mezger G, Schmalohr D, Schneck M, Birbaumer N (2006). Evidence of cortical learning in vegetative state. J Neurol.

[B31] Owen AM, Coleman MR, Boly M, Davis MH, Laureys S, Pickard JD (2006). Detecting awareness in the vegetative state. Science.

[B32] Nachev P, Husain M (2007). Comment on "Detecting awareness in the vegetative state". Science.

[B33] Fins J, Shiff ND (2006). Shades of gray: new insights into the vegetative state. Hastings Cent Rep.

[B34] Panksepp J (1998). Affective Neuroscience, The Foundations of Human and Animal Emotions.

[B35] Panksepp J (2005). Affective consciousness: Core emotional feelings in animals and humans. Conscious Cogn.

[B36] Denton D (2006). The Primordial Emotions: the Dawning of Consciousness.

[B37] Panksepp J (1998). The periconscious substrates of consciousness: Affective states and the evolutionary origins of the SELF. J Consciousness Stud.

[B38] Panksepp J, Normansell LA, Cox JKF, Siviy S (1994). Effects of neonatal decortication on the social play of juvenile rats. Physiology & Behavior.

[B39] Merker B (2007). Consciousness without a cerebral cortex: A challenge for neuroscience and medicine. Behav Brain Sci.

[B40] Shewmon DA, Holmes GL, Byrne PA (1999). Consciousness in congenitally decorticate children: developmental vegetative state as self-fulfilling prophecy. Dev Med Child Neurol.

[B41] Panksepp J (2005). On the embodied neural nature of the core emotional affects. J Consciousness Stud.

[B42] LeDoux JE (1996). The Emotional Brain.

[B43] Rolls ET (2005). Emotions Explained.

[B44] Liotti M, Panksepp J, Panksepp J (2004). On the neural nature of human emotions and implications for biological psychiatry. Textbook of Biological Psychiatry.

[B45] Narvaez D Triune ethics: The neurobiological roots of our multiple moralities. New Ideas Psychol.

[B46] Verheijde JL, Rady MY, McGregor J (2007). Recovery of transplantable organs after cardiac or circulatory death: Transforming the paradigm for the ethics of organ donation. Philos Ethics Humanit Med.

[B47] Evans WD (2007). Seeking an ethical and legal way of procuring transplantable organs from the dying without further attempts to redefine human death. Philos Ethics Humanit Med.

[B48] Veatch RM (2004). Abandon the dead donor rule or change the definition of death?. Kennedy Inst Ethics J.

[B49] President's Council on Bioethics. Session 1 The draft white paper on the determination of death. Discussion by Council Members.

